# Immune-Endocrine Cross-Talk in Reproductive Biology and Pathology

**DOI:** 10.1155/2014/856465

**Published:** 2014-04-24

**Authors:** António M. Galvão, Graça M. Ferreira-Dias, Anna Chełmonska-Soyta, Izabela Wocławek-Potocka, Dariusz J. Skarżyński

**Affiliations:** ^1^Department of Reproductive Immunology and Pathology, Institute of Animal Reproduction and Food Research of PAS, 10-748 Olsztyn, Poland; ^2^Department of Morphology and Function, Faculty of Veterinary Medicine, University of Lisbon, 1300-477 Lisbon, Portugal; ^3^Laboratory of Reproductive Immunology, Department of Experimental Therapy, Institute of Immunology and Experimental Therapy of PAS, 53-114 Wroclaw, Poland

Regulation of the reproductive function in mammals is a very complex biological process, tightly coordinated by the interplay between hypothalamic-pituitary axis and gonads. A myriad of factors regulates both estrous cycle and pregnancy, through the in situ activation of molecular pathways that coordinate subsequent cellular changes. As a result, morphologic and functional changes in reproductive organs ensure the adequate environment for fertilization and pregnancy establishment. Immune system and local immune regulations earn a prominent role in the coordination of those processes. Indeed, the immune-endocrine cross talk regulates several functions at the ovary, such as ovulation, luteal growth/regression, and at the oviduct it ensures fertilization. Besides, it enables maternal recognition of pregnancy in some species, as well as early embryo development and implantation in the uterus. The nature of the links and consequences of mutual interactions between the immune system and physiologic and/or pathologic processes during pregnancy are complex and still not well recognized. A proper fetomaternal immune-endocrine cross-talk during early pregnancy is fundamental for reproductive success. The subject is vastly discussed in this special issue.

Lipids are determinant for reproduction.  I. Wocławek-Potocka and collaborators review the recent progresses in lysophosphatidic acid (LPA) signaling research relevant to human and ruminant reproduction, aiming at the cow as a relevant model to study LPA influence on human reproductive performance. E. G. Prates and coauthors characterize the current knowledge on lipids and fatty acid metabolism during oocyte maturation and their implications on fertilization and embryo development, especially in the pig. Specifically, the possibility of using chemical molecules to modulate the lipid content of oocytes and embryos to improve cryopreservation, as well as their biological effects during different development stages, is debated.

A. Rapacz-Leonard and coauthors revisit the role of the immune system in fetal-maternal cross-talk in humans, cattle, and horses. This review examines physiologic aspects of pregnancy, pathological pregnancy, and parturition. It is suggested that, in horses and cows, the expression of paternal antigens by invading trophoblast cells may modulate the maternal immune system and prepare it for rapid rejection of fetal membranes at parturition. Another study from T. Maj and coworkers address the regulation of peripheral tolerance of maternal immune system during pregnancy establishment. An antigen presentation effectiveness of splenic antigen presenting cells (APCs) derived from pregnant and pseudopregnant mice in* in vitro* conditions was assessed. They showed that CD80 and CD86 costimulatory molecules regulate CD4^+^, T lymphocyte proliferation, and cytokine response in cocultures with antigen-presenting cells derived from pregnant or pseudopregnant mice. Nevertheless, the implications of these changes await further investigation. Finally, immunosuppressive M2-like activities of macrophages at the fetal-maternal interface are required for the maintenance of immunological homeostasis during pregnancy. The findings of D. Rami et al. strengthen the key role of macrophages in counteracting inflammatory stimuli during pregnancy, suggesting procalcitonin as a possible new marker of M1-like macrophages.

Three studies address placenta secretory activity. C. Mannelli et al. showed that human endometrial cells can retain bisphenol, thus reducing the availability of this chemical for the placenta. They highlight the importance of* in vitro* models in reproducing the effect of environmental chemicals on complex systems, such as the fetomaternal interface. The capacity of feline placenta to synthesize prostaglandin F2*α* in a time-dependent manner and the specific enzymatic pathways were reported by M. J. Siemieniuch et al. In the study of Siwetz and collaborators was demonstrated that human placenta is a source of fractalkine, which is expressed in the syncytiotrophoblast, and can be released into the maternal circulation by constitutive metalloprotease-dependent shedding.

A study in rats determined the effect of azithromycin on LPS-induced pregnancy loss. A. Er concluded that azithromycin may be useful for the treatment of infection- or endotoxemia-dependent pregnancy loss.

One manuscript deals with the functional link between interleukins and ovarian steroids on the stimulation of prostaglandins production by endometrial cells from mares. The article by A. Z. Szóstek and collaborators suggests that this interaction could be one of the mechanisms responsible for local orchestrating events in the mare endometrium during the estrous cycle and early pregnancy including implantation.

The manuscript by E. Aliagas et al. addresses the expression of ectonucleotidases CD39 and CD73 in human endometrial cancer, namely, type I endometrioid adenocarcinomas and type II serous adenocarcinomas. This study strengthens the involvement of the adenosinergic system in cancer, emphasizing the relevance of ectonucleotidases as emerging therapeutic targets in oncology.

The ability of proinflammatory cytokines and endotoxins in circulation to affect LH secretion from the pituitary is demonstrated* in vivo *in ewes by K. Haziak et al.

Y. Fan and coauthors tested the hypothesis that intraprostatic 5*α*-dihydrotestosterone exerts an effect on T-cell recruitment by benign prostatic hyperplasia epithelial cells. This study shows that intraprostatic 5*α*-dihydrotestosterone might regulate the inflammatory response induced by human prostatic epithelial cell, via modulation of chemokine (C-C motif) ligand 5 secretion.

Gathering together the present original manuscripts, we do hope to give a fruitful contribution for the knowledge of immune-endocrine interactions regulating reproductive function and dysfunctions in females and males. Understanding the complexity of molecular and cellular pathways linking inflammation to vascular and nonvascular tissue remodeling, secretory function and dysfunction in reproductive organs are vital. Contributing for the identification of potential molecular targets for future pharmacological interventions in prevention and treatment of infertility is an asset.

*António M. Galvão* *António M. Galvão* *Graça M. Ferreira-Dias* *Graça M. Ferreira-Dias* *Anna Chełmonska-Soyta* *Anna Chełmonska-Soyta* *Izabela Wocławek-Potocka* *Izabela Wocławek-Potocka* *Dariusz J. Skarżyński* *Dariusz J. Skarżyński*

